# Correlation between CT-derived fractional flow reserve and myocardial strain in ischemic heart disease patients with single coronary artery stenosis assessed based on CCTA

**DOI:** 10.3389/fcvm.2025.1525807

**Published:** 2025-01-23

**Authors:** Ruichen Ren, Wenting Li, Qingyuan Zhao, Chengcheng Qi, Xiaoxue Zhang, Mingyu Peng, Duwang Su, Pei Han, Yang Zhang

**Affiliations:** Department of Radiology, Qilu Hospital of Shandong University, Jinan, China

**Keywords:** coronary computed tomography angiography, coronary artery disease, ischemic heart disease, fractional flow reserve, strain

## Abstract

**Purpose:**

We aimed to investigate the correlation between CT-derived fractional flow reserve (CTFFR) and myocardial strain in patients with single coronary artery stenosis and to investigate the diagnostic value of CTFFR in identifying impaired myocardial strain.

**Methods:**

We selected 89 patients, categorized into three groups based on the affected coronary artery: 36 with left anterior descending (LAD), 23 with left circumflex (LCX), and 30 with right coronary artery (RCA) stenosis, along with 25 healthy controls. We investigated correlations between CTFFR and both global and regional myocardial strain parameters. Additionally, we assessed the ability of the CTFFR to detect impaired myocardial strain in these patients.

**Results:**

In this study, no significant difference was found in overall myocardial strain between the patient and control groups. However, regional longitudinal strain (LS) and circumferential strain (CS) in the myocardial areas supplied by stenotic coronary arteries was significantly lower in each patient group compared to the others (*P* < 0.001). The CTFFR exhibited a strong negative correlation with both regional and global myocardial strain, with a stronger association for regional strain. Particularly in group LAD, CTFFR in optimal diastole phase (CTFFR-D) was negatively correlated with Endo-LS (*r* = −0.66, *P* < 0.001). Receiver operator characteristic curve (ROC) analysis indicated that CTFFR were effective in diagnosing impaired myocardial strain, particularly LS.

**Conclusion:**

There is a strong correlation between CTFFR, which is a functional measure for assessing coronary artery stenosis, and myocardial strain. CTFFR can identify impaired myocardial strain and can be used as an indirect indicator of myocardial ischemia.

## 1 Introduction

The main cause of ischemic heart disease is atherosclerosis (1, 2), on the basis of which the lumen narrows or even occludes as the extent of the lesion increases, leading to long-term myocardial ischemia, myocyte injury, necrosis, and myocardial fibrosis, resulting in structural changes in the heart and a decline in cardiac function (3, 4). Conventional Coronary Computed Tomography Angiography (CCTA) provides only anatomical information about the coronary arteries and often overestimates the degree of stenosis, leading to unnecessary invasive coronary angiography (5). CTFFR has emerged as a computational fluid dynamics method for assessing the severity of functional coronary stenosis without the need for additional radiation and loading medications (6, 7). CCTA-derived myocardial strain can identify early changes in myocardial function when conventional cardiac function markers are still in the normal range (8). The results are highly consistent with those of echocardiography and cardiac magnetic resonance imaging (CMR) (9). It has now been shown that patients with coronary artery stenosis present with early impaired cardiac function (10, 11). However, the correlation between functional coronary stenosis and impaired myocardial function has not been studied. Therefore, we chose CTFFR as a functional index of coronary artery to investigate the correlation with myocardial strain.

## 2 Methods

### 2.1 Study population

This study retrospectively analyzed patients with diagnosed coronary heart disease with single coronary stenosis who underwent CCTA between March 2023 and March 2024 at Qilu Hospital of Shandong University. Coronary stenosis was visually assessed by a cardiovascular radiologist (Y.Z.) with over 20 years of experience in cardiovascular imaging. Stenosis was defined as the presence of luminal narrowing caused by coronary plaque observed on CCTA. This study was approved by our institutional review board (Ethical Approval No. KYLL-202208-057) and the informed consent requirement was waived due to its retrospective nature. We grouped the patients according to the location of the stenosis into LAD group, LCX group, RCA group.

The inclusion criteria were: (1) patients diagnosed with single coronary stenosis; (2) clinically confirmed subjects aged between 30 and 80 years; and (3) no wall motion disorder on echocardiography. The exclusion criteria were: (1) a history of myocardial infarction (MI) or Q-waves on ECG; (2) known MI, congestive heart failure, heart valve disease, or structural heart disease; (3) atrial fibrillation; (4) arrhythmias; (5) history of coronary intervention, including coronary artery bypass grafting (CABG) and/or percutaneous coronary intervention (PCI); and (6) poor CCTA image quality that cannot be assessed.

### 2.2 CT scanning protocol and postprocessing

All examinations were performed using a third-generation dual-source CT scanner (SOMATOM Force CT, Germany). A retrospective ECG-gated scanning mode was used to control for cardiac-related motion artifacts. Scanning range: starting 1 cm below the tracheal eminence and extending down to the diaphragmatic surface of the heart, covering all coronary arteries. Scanning parameters were as follows: tube voltage, 70–120 kV; tube current was automatically adjusted to the patient's size by an automatic exposure control system (CARE Dose4D, Siemens Healthineers, Germany). Nonionic contrast agent (Iopromide 370, Bracco) in a dosage of 0.7 ml/kg body weight at 5 ml/s was injected by double-barrel high-pressure syringe following with 40 ml sanitary saline at the same rate. The CT system automatically reconstructed the data for optimal diastole and optimal systole. We reconstructed 20 phases in 5% steps of the R-R interval within the full window. The data construction section thickness was 0.75 mm, with an increment of 0.5 mm, and the reconstruction kernel was Bv40 heart view smooth.

### 2.3 Assessment of the CTFFR of coronary artery

**Figure 1:**
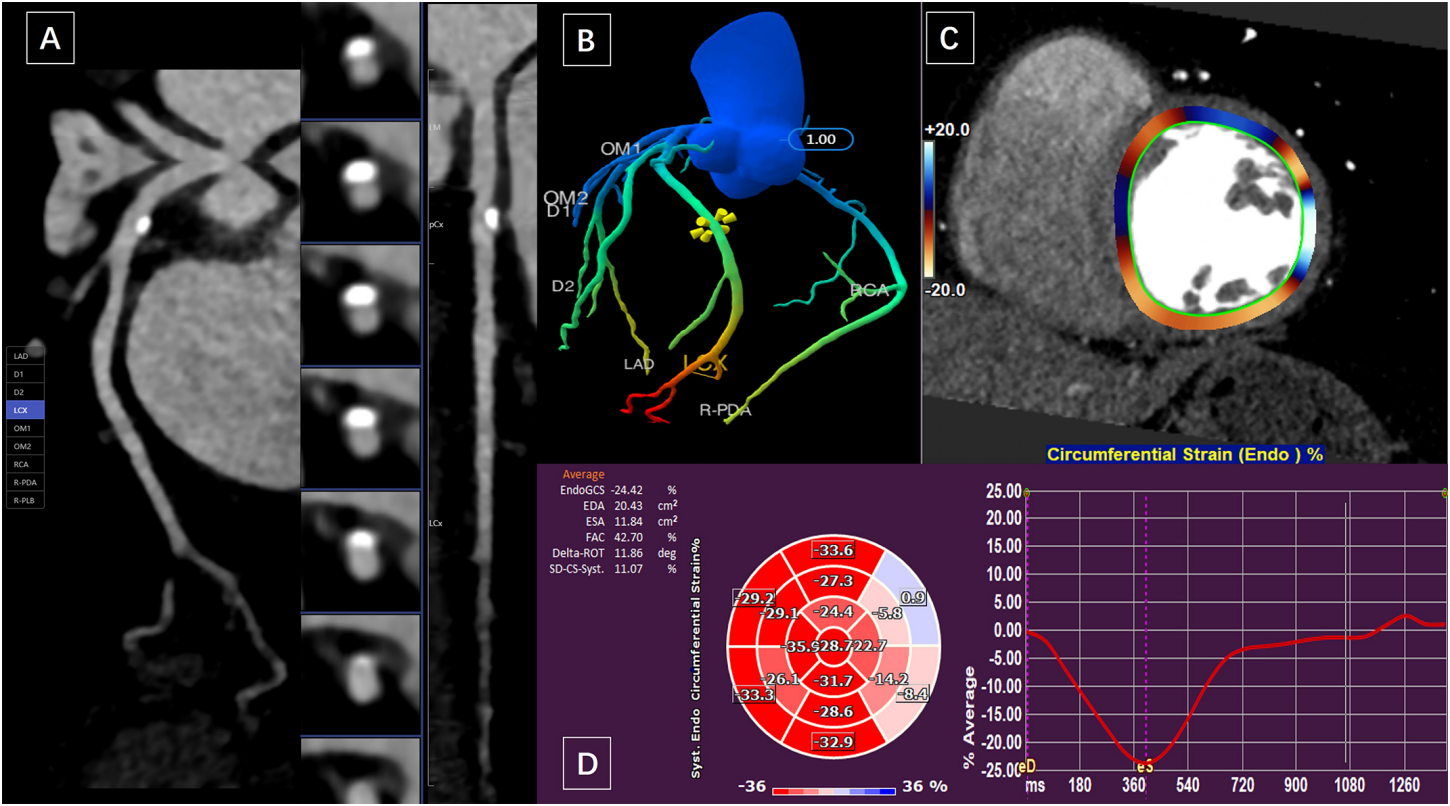
A 59-year-old woman presented with calcified plaque in the proximal wall of the LCX and moderate luminal stenosis. **(A)** Curved planar reformation of CCTA showed calcified plaque in LAD with moderate luminal stenosis. **(B)** The CTFFR tree with numerical markers. **(C)** and **(D)** measurements of circumferential strain of the left ventricular endocardium in short-axis views.

The optimal systolic and diastolic images were transmitted to the Shukun Artificial Intelligence Coronary Diagnostic Aid (skCT-FFR version v0.7.1, Beijing, China) to obtain CTFFR values. This software combines CCTA images with computational fluid dynamics to determine coronary pressures and flow velocities, generating color-coded 3D coronary trees with numerical values (Figure 1). CTFFR values were measured approximately 2 cm distal to the plaque in optimal systolic (CTFFR-S) and diastolic phase.

### 2.4 Computed tomography feature tracking analysis

Raw multiphasic CCTA images were imported into commercially available post-processing software (Medis Suite version 4.0, Leiden, the Netherlands). The entire measurement process similar to zhang et al. (12) involves five steps: (1) evaluating CCTA image quality; (2) reconstructing two-dimensional cine images, including two-, three-, and four-chamber long-axis and stacked short-axis views; (3) manually drawing the endocardial and epicardial contours at the end-diastolic and end-systolic phases; (4) automatically tracking left ventricular (LV) myocardial motion throughout the entire cardiac cycle; and (5) confirming the feature-tracking quality and adjusting the end-systolic location. Longitudinal strain (LS) was derived from long-axis (LAX) cine images, while circumferential strain (CS) and radial strain (RS) were derived from both LAX and short-axis (SAX) cine images. The following strain parameters were derived: (1) global longitudinal strain (GLS) and global circumferential strain (GCS) of the endocardium and myocardium, and global radial strain (GRS) of the myocardium; and (2) territory strain based on the American Heart Association 16-segment model. Segments 1, 2, 7, 8, 13, and 14 were defined as the territory supplied by the left anterior descending artery (LAD), segments 5, 6, 11, 12, and 16 as the territory supplied by the left circumflex artery (LCX) (Figure 1), and segments 3, 4, 9, 10, and 15 as the territory supplied by the right coronary artery (RCA). The segmental strain of the endocardium and myocardium were averaged. In addition, obtaining volumetric and functional parameters of the left ventricle: LV end-diastolic volume (LVEDV), LV end-systolic volume (LVSDV), ejection fraction (EF). All images were evaluated and measured by two observers who were blinded to the patients' information.

### 2.5 Statistical analysis

The distribution normality of continuous data was assessed using the Shapiro-Wilk test. Data was expressed as means ± standard deviations for normally distributed variables, medians (interquartile ranges) for non-normally distributed variables, and frequencies (percentages) for categorical variables. Comparisons between two groups utilized independent sample Student's *t*-tests for normal data and Mann-Whitney *U* tests for non-normal data. Chi-square tests were employed for categorical variables. Clinical characteristics and CT findings across different coronary artery groups were analyzed using one-way ANOVA with Bonferroni *post hoc* tests or the Kruskal-Wallis *H* test. Pearson's correlation was used for normally distributed variables, while Spearman's correlation was applied for non-normally distributed data. Diagnostic performance of stenosis severity and CTFFR for strain was evaluated using ROC analysis. Area under curve (AUC) comparisons followed the method of DeLong et al. (13). Repeatability between observers was assessed with the Bland-Altman test in 20 randomly selected patients. Statistical analysis was conducted using SPSS 26.0 and GraphPad Prism 10.0.2, with significance set at *P* < 0.05.

## 3 Results

### 3.1 Clinical characteristics of the study population

**Figure 2:**
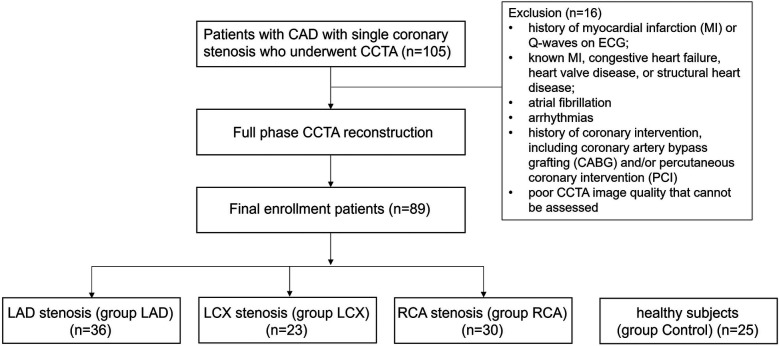
Study flow chart.

A total of 89 patients were included. Among them, 36 patients were diagnosed with LAD stenosis, 23 patients were diagnosed with LCX stenosis, 30 patients were diagnosed with RCA stenosis. Additionally, 25 healthy subjects were included as controls (Figure 2 Study flow chart). The patients' demographics and clinical characteristics were summarized in Table 1. Patients with coronary stenosis were predominantly male who were older and had a higher systolic blood pressure. There were no statistically significant differences between the all groups with respect to BMI and smoking history, and no statistically significant difference in the hypertension, diabetes mellitus and hyperlipidemia among the patients' groups.

**Table 1:** Clinical characteristics of the study population.

Variable	Group LAD (*n* = 36)	Group LCX (*n* = 23)	Group RCA (*n* = 30)	Control (*n* = 25)	*P* value
Male (*n*, %)	24 (66.7)	13 (56.5)	17 (56.7)	11 (44)	0.392
Age (years)	56 ± 9	59 ± 10	57 ± 11	51 ± 7**	0.013*
BMI (kg/m²)	25.3 ± 4	25.0 ± 3	25.5 ± 2	23.3 ± 2	0.063
Hypertension (*n*, %)	20 (55.6)	11 (47.8)	18 (60)	–	0.683
SBP	141 ± 16	137 ± 20	140 ± 16	126 ± 13	0.003*
DBP	82 ± 8	84 ± 10	84 ± 10	83 ± 10	0.813
Diabetes mellitus (*n*, %)	11 (30.6)	5 (21.7)	5 (16.7)	–	0.413
Smoking history (*n*, %)	10 (27.8)	7 (30.4)	8 (26.7)	4 (16)	0.667
Hyperlipidemia (*n*, %)	13 (36.1)	6 (26.1)	9 (30)	–	0.713
CCTA heart rate (beats/min)	64 ± 12	69 ± 14	66 ± 12	71 ± 15	0.248
Total cholesterol (mmol/L)	5	4.7	4.8	–	–
Creatinine (umol/L)	65.3	65.7	70	–	–
Creatine kinase (U/L)	66.4	78	79	–	–
Troponin I (ng/L)	9.1	4.1	11.4	–	–

LAD, left anterior descending; LCX, left circumflex; RCA, right coronary artery; BMI, body mass index; SBP, systolic blood pressure; DBP, diastolic blood pressure; CCTA, coronary computed tomography angiography.

* *P* < 0.05 between groups.

** *P* < 0.05 compared with group LCX.

### 3.2 Comparison of CTFFR and CT strain in the study groups

The patients' CT parameters of CTFFR and CT strain were shown in Table 2. The CTFFR of diastole and systole in the patient groups were significantly reduced comparing to the control group (*P* < 0.001). There were no statistically significant differences in global strain among groups.

**Table 2:** Cardiac CT parameters of the study population.

Observation indicators	Group LAD (*n* = 36)	Group LCX (*n* = 23)	Group RCA (*n* = 30)	Control (*n* = 25)	*P* value
CTFFR					
CTFFR-D	0.75 ± 0.11	0.80 ± 0.06	0.80 ± 0.10	0.90 ± 0.04**^,^ ***^,^ ****	<0.001*
CTFFR-S	0.75 ± 0.11	0.80 ± 0.06	0.80 ± 0.08	0.90 ± 0.04**^,^ ***^,^ ****	<0.001*
CT strain (%)					
Global					
EndoGLS	−25.8 ± 3.7	−27.0 ± 3.3	−27.1 ± 3.7	−28.1 ± 3.1	0.101
MyoGLS	−23.3 ± 2.8	−23.8 ± 3.0	−24.7 ± 2.3	−25.0 ± 2.6	0.058
EndoGCS	−30.6 ± 5.0	−31.8 ± 5.1	−31.9 ± 5.1	−32.7 ± 4.6	0.437
MyoGCS	−26.0 ± 5.6	−23.5 ± 6.7	−24.9 ± 6.7	−25.9 ± 6.7	0.468
GRS	83.5 ± 16.8	87.3 ± 18.6	91.8 ± 16.4	92.2 ± 12.8	0.117
EF	68.5 ± 5.9	69.0 ± 6.3	70.1 ± 4.8	69.5 ± 5.9	0.701
LVEDV	131.5 ± 26.2	126.8 ± 29.2	134.0 ± 34.2	127.8 ± 31.4	0.802
LVESV	42.8 ± 13.9	41.0 ± 14.3	45.0 ± 14.4	40.0 ± 2.9	0.571
Territory-LS					
LAD	−18.7 ± 3.5	−25.4 ± 4.5**	−23.1 ± 2.3**	−25.3 ± 2.6**	<0.001*
LCX	−23.1 ± 4.0	−19.2 ± 3.3**	−23.8 ± 3.4***	−25.5 ± 4.3***	<0.001*
RCA	−22.0 ± 3.2	−20.16 ± 3.7**	−22.0 ± 3.9***	−22.1 ± 5.3***	0.269
Territory-CS					
LAD	−24.4 ± 6.5	−23.0 ± 6.7	−25.8 ± 7.5	−26.1 ± 7.1	0.397
LCX	−21.0 ± 7.2	−22.7 ± 6.6	−23.6 ± 7.3	−24.2 ± 6.3	0.590
RCA	−25.5 ± 5.9	−22.9 ± 8.3	−25.1 ± 7.2	−26.5 ± 8.5	0.056
Territory-RS					
LAD	55.1 ± 12.7	62.1 ± 18.5	60.5 ± 16.7	74.9 ± 38.0**	0.005*
LCX	55.8 ± 23.3	53.7 ± 14.2	57.9 ± 18.1	63.8 ± 19.2	0.189
RCA	53.4 ± 18.0	57.5 ± 15.6	55.0 ± 17.5	64.9 ± 11.0	0.113

CTFFR, computed tomography fractional flow reserve; CTFFR-D, computed tomography fractional flow reserve in optimal diastole phase; CTFFR-S, computed tomography fractional flow reserve in optimal systole phase; LAD, left anterior descending; LCX, left circumflex; RCA, right coronary artery; EndoGLS, global longitudinal strain of endothelia; MyoGLS, global longitudinal strain of myocardium; EndoGCS, global circumferential strain of endothelia; MyoGCS, global circumferential strain of myocardium; GRS, global radial strain; EF, ejection fraction; LVEDV, left ventricular end diastolic volume; LVESV, left ventricular end systolic volume; LS, longitudinal strain; CS, circumferential strain; RS, radial strain.

* *P* < 0.05 between groups.

** *P* < 0.05 compared with groups LAD.

*** *P* < 0.05 compared with groups LCX.

**** *P* < 0.05 compared with groups RCA.

However, LS regional strain was significantly decreased (absolute value, same below) in the donor regions of stenotic coronary artery in group LAD and LCX (*P* < 0.001). RS regional strain was decreased in the LAD donor region in group LAD (*P* = 0.005).

### 3.3 Comparison of strain between stenosis coronary territory and non-stenosis coronary territory in the patients' group

**Figure 3:**
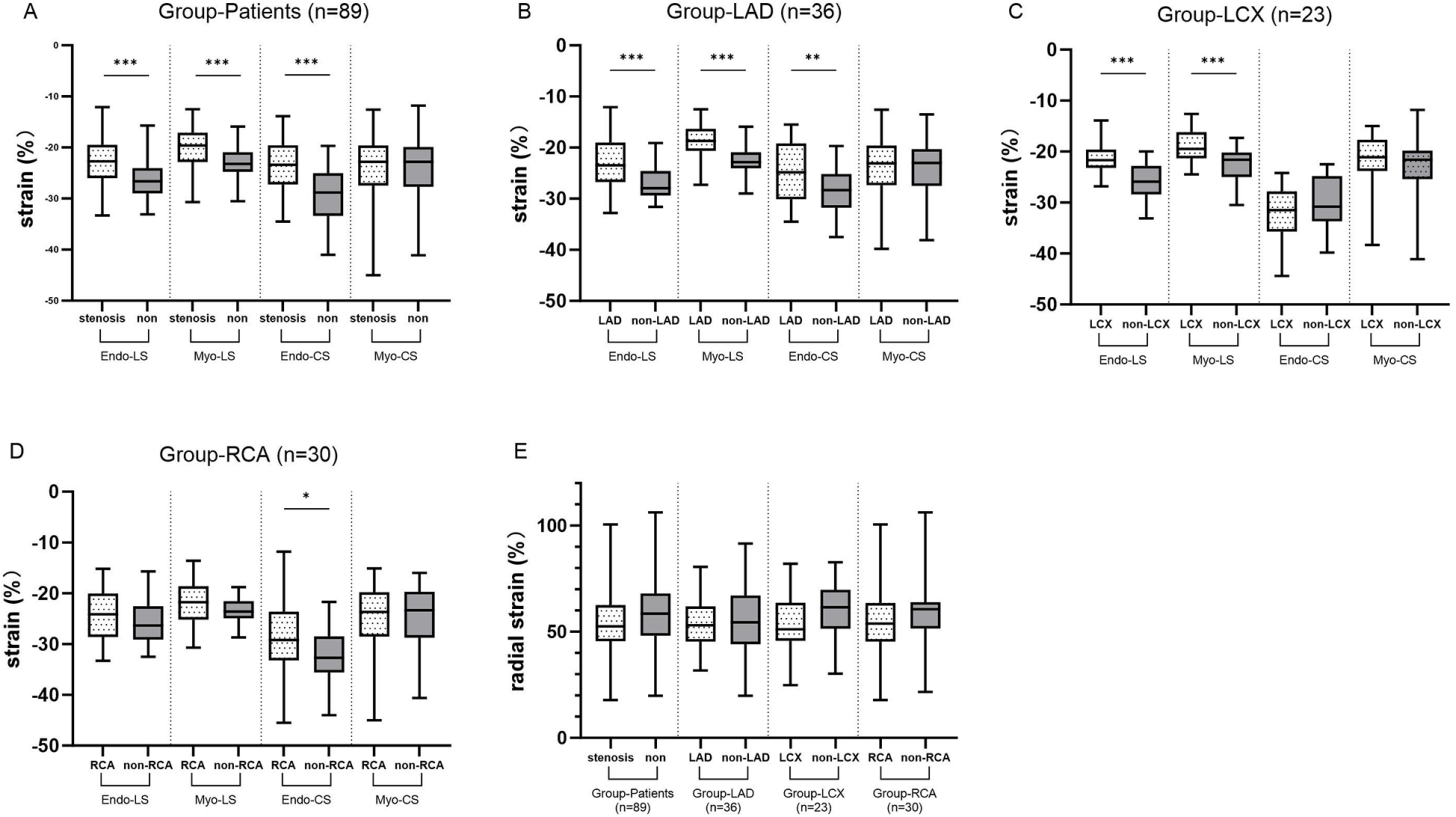
**(A)** The differences in LS and CS between stenotic coronary-supplied myocardium and non-stenotic myocardium in the patient groups. **(B–D)** The differences in LS and CS between stenotic coronary-supplied myocardium and non-stenotic myocardium in the LAD, LCX, and RCA groups, respectively. **(E)** The differences in RS between stenotic coronary-supplied myocardium and non-stenotic myocardium within the patient population as a whole and within each subgroup.

These results are shown in Figure 3. In the Patients group (group LAD+LCX+RCA), Endo-LS (*P* < 0.001), Myo-LS (*P* < 0.001) and Endo-CS (*P* < 0.001) in the stenotic coronary-supplied region were decreased compared with in the non-stenotic coronary-supplied region. Specifically, in the LAD group, Endo-LS (*P* < 0.001), Myo-LS (*P* < 0.001) and Endo-CS (*P* = 0.013) in the LAD-supplied territory were significantly decreased. In the LCX group, Endo-LS (*P* < 0.001) and Myo-LS (*P* < 0.001) in the LCX-supplied area were significantly decreased. In the RCA group, Endo-CS (*P* = 0.044) was significantly decreased in the RCA-supplied myocardium. RS in the stenotic coronary-supplied region was not significantly different from that in the non-stenotic region in group LAD, LCX, RCA and Patients.

### 3.4 Correlations between CTFFR and CT strain in patients group

**Figure 4:**
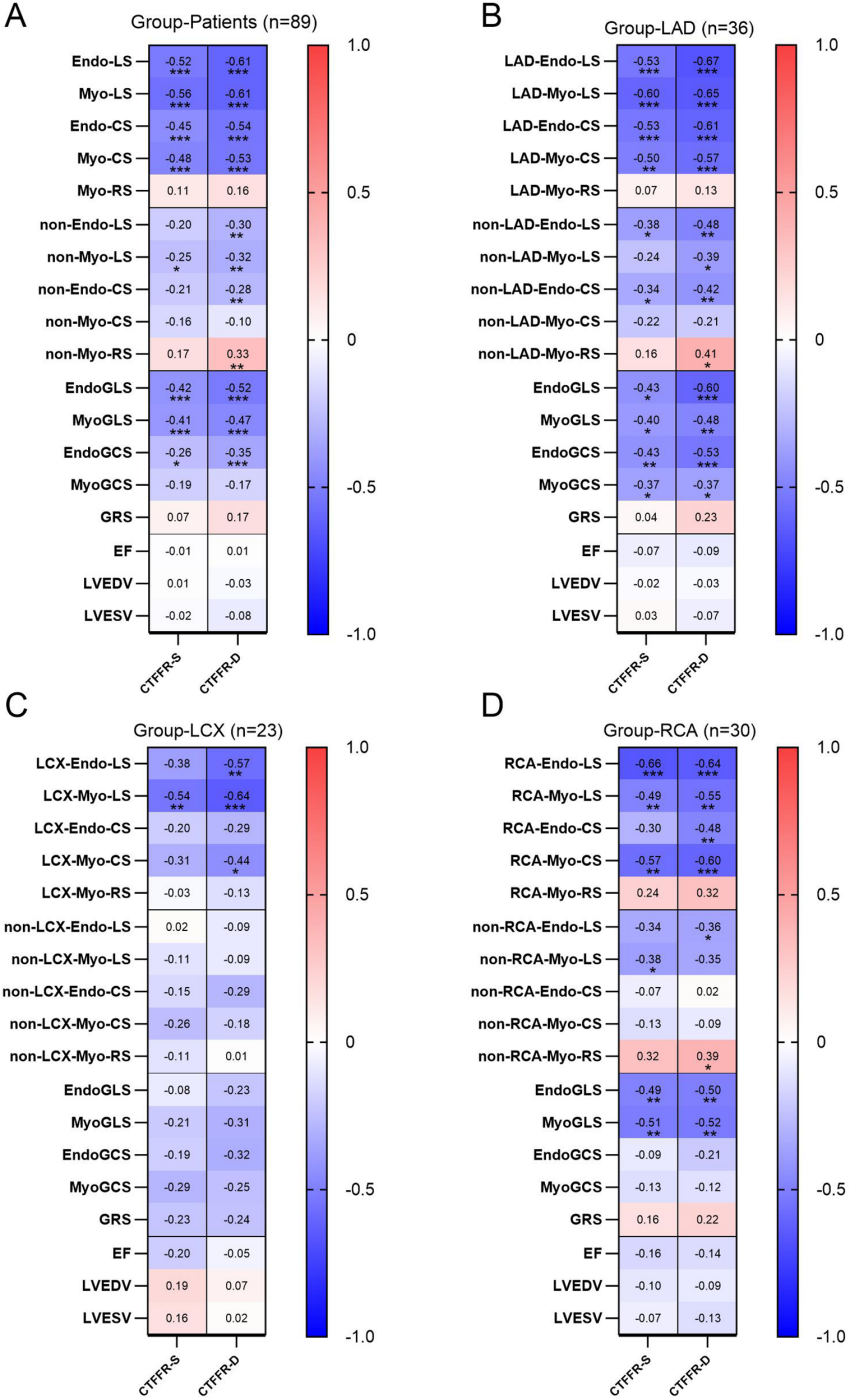
The correlation between CTFFR and strain in the patients group **(A)**, group LAD **(B)**, group LCX **(C)**, and group RCA **(D).**

The correlations between CTFFR and CT strain in group LAD, LCX, RCA and Patients were shown in Figure 4. Overall, CTFFR was negatively correlated with LS, CS of stenotic coronary-supplied (*P* < 0.05) and not significantly correlated with RS. The correlation between CTFFR and regional myocardial strain was greater than that of global strain. The correlation between CTFFR-D and myocardial strain was greater than that of CTFFR-S. There was no significant correlation between CTFFR and ejection fraction and left ventricular volume. In the group LAD, CTFFR and strain in the non-stenotic coronary-supplied also showed a correlation. In the group LCX, CTFFR showed a correlation only with the strain in regions of stenotic coronary-supplied.

### 3.5 Diagnostic performance of the CTFFR to recognize impaired myocardial strain

**Figure 5:**
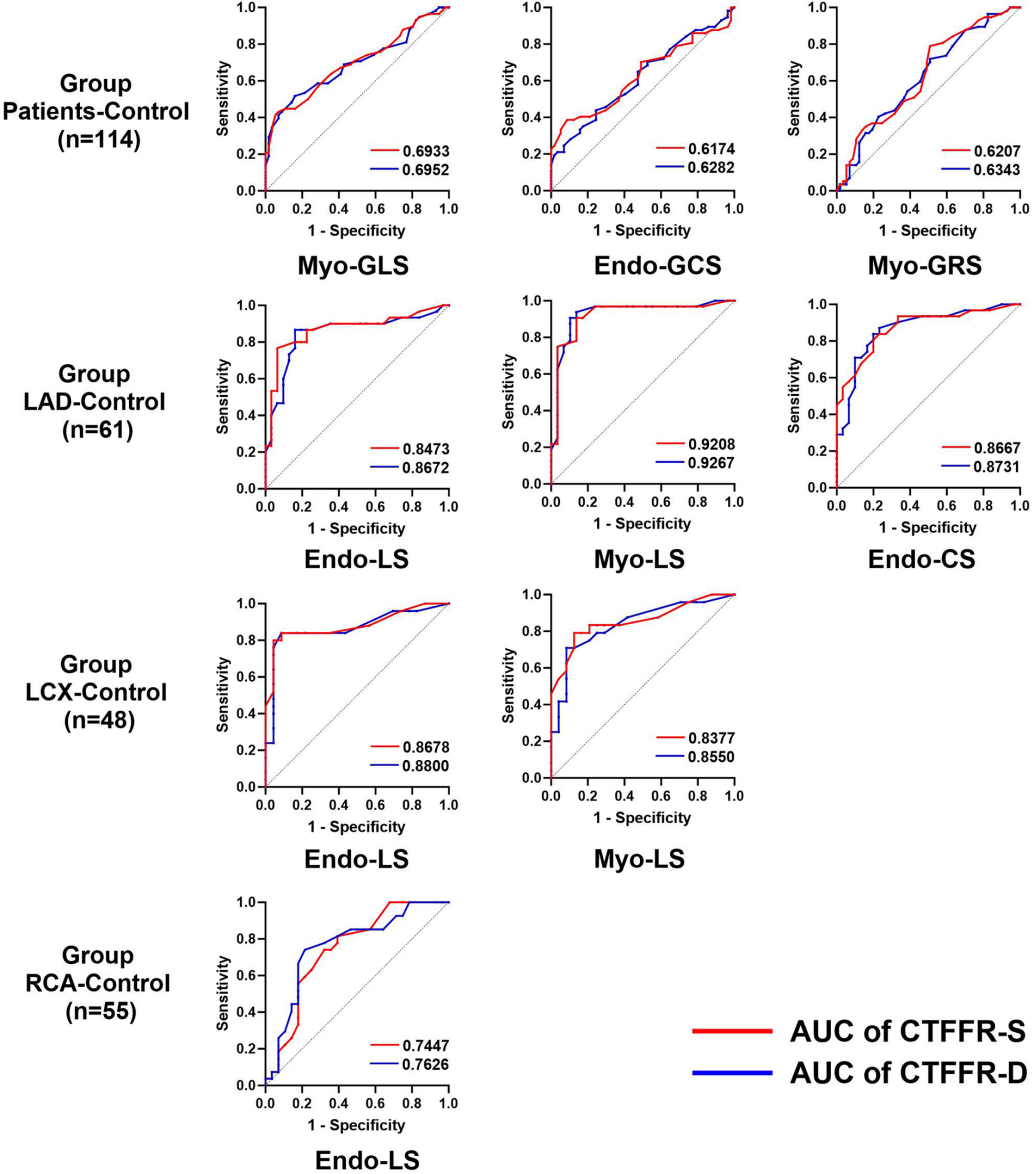
Diagnostic performance of the CTFFR to recognize impaired myocardial strain in group Patients-control, group LAD-control, group LCX-control and group RCA-control.

Since the consensus on normal myocardial strain values has not been established due to differences in post-processing algorithms, we mixed the control group with group Patients, LAD, LCX and RCA. We performed ROC curve using the median of each strain parameter as a diagnostic criterion in order to investigate the diagnostic performance of CTFFR for impaired myocardial strain, respectively. The results (Figure 5) showed that CTFFR-S and CTFFR-D had diagnostic value for impaired Myo-GLS, Endo-GCS, and Myo-GRS in all enrolled populations (*P* < 0.05), and the diagnostic performance was higher for Myo-GLS (AUC of CTFFR-S = 0.6933, AUC of CT-FFR-D = 0.6952). In the LAD-Control group, CTFFR had diagnostic value for impaired Endo-LS, Myo-LS and Endo-CS (*P* < 0.05), with higher diagnostic performance for Myo-LS (AUC of CTFFR-S = 0.9207, AUC of CT-FFR-D = 0.9267). In the LCX-Control group, CTFFR had diagnostic value for Endo-LS and Myo-LS (*P* < 0.05), with higher diagnostic performance for Endo-LS (AUC of CTFFR-S = 0.8678, AUC of CT-FFR-D = 0.8800). In the RCA-Control group, CTFFR had diagnostic value only for Endo-LS (AUC of CTFFR-S = 0.7447, AUC of CT-FFR-D = 0.7626, *P* < 0.05). Overall, CTFFR had the best diagnostic performance for impaired LS compared with CS and RS. CTFFR had the greatest diagnostic value for myocardial strain in the LAD-supplied area compared with the LCX-Control and RCA-Control groups. Supplementary Table 1 provides the specificity, sensitivity, and 95% confidence intervals (CIs) for each ROC analysis performed in the study.

### 3.6 Intra- and interobserver reproducibility of LV global strain

There was good intra- and interobserver agreement in the assessment of global strain in the LV myocardium. The GRS had a relatively low reproducibility (Table 3).

**Table 3:** Intra- and interobserver reproducibility of LV global strain.

Strain	Intra-observer		Inter-observer	
	ICC	CV	ICC	CV
EndoGLS	0.888	17.2%	0.773	14.1%
MyoGLS	0.820	13.6%	0.593	12.5%
EndoGCS	0.852	19.4%	0.629	14.4%
MyoGCS	0.897	21.6%	0.890	19.9%
GRS	0.647	16.8%	0.297	23.3%

ICC, intraclass correlation coefficient; CV, Coefficient of variation.

## 4 Discussion

The major findings of study were as follows: (1) In patients with single coronary stenosis, regional longitudinal strain in stenotic coronary-supplied myocardium was already significantly impaired compared with controls before global strain was significantly reduced. (2) Endocardial and myocardial longitudinal strain and circumferential strain were impaired in stenotic coronary-supplied myocardium compared with non-stenotic coronary-supplied regions. (3) CTFFR was negatively correlated with myocardial strain, and the correlation with regional strain was greater than that with global strain. (4) Using the median strain of patients and normal subjects as a diagnostic criterion, CTFFR have good diagnostic value for myocardial strain, especially for LS.

Ample evidence exists that endothelial dysfunction in resistance coronary vessels is an important contributor to CMD (14). With the development of atherosclerosis, the vascular endothelium becomes dysfunctional, and the vasodilator response to pharmacological and physiological interventions is attenuated. This results in a frank reduction in blood flow (15), leading to myocardial ischemia and a decrease in myocardial diastolic function. CCTA is a widely used modality in clinical practice to screen and detect cardiovascular disease. Conventional CCTA provides only anatomical information about coronary stenosis, while functional information related to the stenosis remains elusive. This leads to an increased referral rate for invasive coronary angiography (ICA) (16, 17). Functional damage to the vessel often precedes structural changes (18). Therefore, we used CTFFR for the assessment of functional stenosis in coronary arteries. CTFFR values demonstrates a high level of agreement with invasively measured FFR, especially from 0.49 to 0.90 [with an average difference of 0.01 (95% CI, 0.21 to −0.24)] (19). When the CTFFR is >0.90, it can rule out hemodynamically significant coronary artery disease. Lesions with an FFR ≤0.80 are recommended for revascularization, while the lesions with an FFR >0.80 are recommended for optimized medical therapy. The interpretation of CTFFR results is consistent with the invasive FFR recommendation (20, 21). Therefore, CTFFR can be an important adjunct to CCTA and is critical for avoiding unnecessary interventions as well as saving costs in the healthcare system.

Although left ventricular ejection fraction (LVEF) is the conventionally used index for assessment of LV function, it largely reflects the pump function rather than the mechanical function. Myocardial strain has been shown to be an effective parameter for assessing mechanical function, and strain can detect subtle changes in myocardial function compared with LVEF, especially regional strain (22, 23). Regional strain has rarely been used in prior studies due to a belief in current echocardiographic guidelines that assessment of regional strain is not as robust and reliable as overall strain (24). We analyzed patients' strain with single coronary artery stenosis. The results showed that longitudinal strain in the region of the stenotic coronary artery supplying myocardium was significantly impaired before global strain was significantly impaired. This was true for the LAD, LCX, and RCA stenosis groups. Other studies have shown that global and regional longitudinal myocardial strain is significantly lower in patients with severe stenoses in the LAD (25). Norum et al. used echocardiography for strain analysis in patients admitted for chest pain and initial negative/slightly elevated troponin, and found that regional strain changes detected severe coronary artery disease (CAD) (26). These confirm the clinical significance of assessing local myocardial strain.

Our study demonstrated that CTFFR was correlated well with regional and global strain, especially with longitudinal and circumferential strain. The ROC curves further showed that CTFFR had a good diagnostic performance for identifying impaired strain. The LV heart wall comprises three layers: the oblique endocardial, the circular mid-myocardial, and the oblique epicardial layer. Since the endocardial-layer fibers are mainly oriented in the longitudinal direction, and endocardial myocardial fibers account for 15% of cardiomyocytes yet are required to produce more than 50% of the ejection fraction in myocardial contraction (27). Longitudinal strain is therefore most impaired in coronary stenosis and is of high value for disease risk assessment and prognostic prediction. Global longitudinal peak strain at rest evaluated using 2-dimensional echocardiography is an independent predictor of severe coronary disease and significantly improves the diagnostic performance of exercise tests (28). 2D STE-derived longitudinal strain may help to identify a significant proportion of patients with critically narrowed LAD and subclinical regional LV dysfunction not appreciated by visual assessment (25). In contrast, short-axis function assessed by circumferential shortening is largely determined by circumferential fiber contraction. It has been shown that circumferential strain rate predicts ventricular remodeling in patients with high-risk myocardial infarction and that preserved circumferential function contributes to the suppression of ventricular enlargement after myocardial infarction (29). In this study, radial strain of myocardium in the donor area of stenotic coronary arteries was not significantly different from that of myocardium in the non-donor area, and CTFFR was not significantly correlated with radial strain. The lower reproducibility of radial strain measurements may account for the variability not observed in this study. It may be caused by the quantification of the radial strain, which depends on the simultaneous motion of the endocardium and epicardium. The density contrast at the epicardial border is less pronounced than at the endocardial border. Moreover, there are more myocardial trabeculae in the apical region. The compression and drainage of blood from the trabeculae at the end of systole alter the voxel appearance in this region, making accurate tracking challenging (23).

Previous studies have reported significant correlations between coronary stenosis degree, CAD-RADS scores and myocardial strain. Gu et al. (30) used CT strain to assess local LV strain in patients with LAD stenosis and showed that strain in the LAD region decreased with increasing stenosis severity. Shi et al. (31) used CT feature tracking to assess myocardial strain in patients with different CAD-RADS and showed that as the CAD-RADS level increased, the GCS, GLS and GRS of the left ventricle based on CT gradually decreased. Our findings are generally consistent with these, both showing a progressive decrease in the absolute values of GLS, GCS, and GRS with decreasing CTFFR. In this study, no significant difference was found between diastolic and systolic CTFFR among the groups, but heat maps, ROC curves and delong tests showed higher correlation and diagnostic value of CTFFR-D with myocardial strain. This may be because diastole, which comprises the majority of the cardiac cycle, is optimal for coronary image acquisition and reduces motion artifacts (32). Additionally, the vessel lumen may appear more “normal” during diastole, making diastolic FFR a more suitable approach for assessing the hemodynamic significance of myocardial bridging (33). Lv et al. (34) analyzed CTFFR in diastole and systole using FFR as the reference standard. Their findings indicated that CTFFR-D had higher diagnostic accuracy and sensitivity, with a smaller absolute difference between CTFFR-D and FFR at FFR ≤ 0.7. Therefore, we recommend using diastolic CTFFR for the hemodynamic assessment of coronary arteries.

In this study, we used the median myocardial strain in patients and normal group as a diagnostic criterion. The absolute values less than the median were considered to be impaired strain. This is because there are no standardized criteria for the normal range of myocardial strain for CT tracing. Some of the studies on the range of myocardial strain in normal populations have shown different results because of differences in calculation software and measurement methods. McVeigh et al. (35) developed an automated technique, CT SQUEEZ, to measure localized endocardial strain in patients with normal left ventricular function using 4D-CT cine images, and found that the average endocardial strain values in the basal, mid, and apical left ventricular segments were (−32 ± 1)%, (−33 ± 1)%, and (−36 ± 1)%, respectively, with the absolute value of strain increasing from the basal to the apical segment. Li et al. (36) analyzed CT myocardial strain in 87 normal subjects, and GRS of the left ventricle in normal subjects was (74.5 ± 15.2)%, and GCS was (−22.7 ± 3.0)%, and GLS was (−26.6 ± 3.2)%. In a study of CT strain values of four cardiac chambers in 101 healthy adults, Yang et al. (37) found that the normal ranges of GLS, GCS, and GRS in the left ventricle were −20.2 ± 2.7%, −27.9 ± 4.1%, and 49.4 ± 12.1%, respectively. More consistently, however, healthy males have lower longitudinal and circumferential strain with less negative value (37, 38). In contrast, they have higher radial strain with a significant age-dependent positive correlation (36). The reason may be that age-related left ventricular stiffness and decreased diastolic function need to be compensated by increased systolic wall thickness (39).

Our study demonstrated significant correlations between CTFFR and myocardial strain, particularly longitudinal and circumferential strain. However, our patient population included individuals with hypertension and diabetes mellitus, which could introduce variability in the results. Furthermore, CTFFR was analyzed as a functional metric independent of anatomical stenosis severity. While these comorbidities may influence myocardial strain and coronary physiology, our findings suggest that CTFFR provides valuable functional information beyond anatomical assessments. Future studies with larger, stratified cohorts are needed to further explore the interplay between CTFFR, strain, and specific comorbid conditions.

This study has several limitations. First, the retrospective design and small data sample from a single center limit the generalizability of the conclusions. Second, the patients were not validated by CAG, as invasive testing is not appropriate for patients without a history of myocardial infarction. Third, the heart rates of some patients during CCTA examination were higher than 60, potentially influencing strain assessment.

## 5 Conclusion

Our study demonstrated a strong correlation between CTFFR and myocardial strain. In patients with a single coronary artery stenosis, the longitudinal strain and circumferential strain of the myocardium corresponding to the stenotic coronary artery were significantly reduced in absolute values compared to the non-stenotic region. As an index for assessing functional stenosis of coronary arteries, CTFFR can be used to indirectly reflect impaired myocardial function in the corresponding blood supply region.

## Data Availability

The raw data supporting the conclusions of this article will be made available by the authors, without undue reservation.
