# Corrigendum: Correlation between CT-derived fractional flow reserve and myocardial strain in ischemic heart disease patients with single coronary artery stenosis assessed based on CCTA

**DOI:** 10.3389/fcvm.2025.1574571

**Published:** 2025-02-21

**Authors:** Ruichen Ren, Wenting Li, Qingyuan Zhao, Chengcheng Qi, Xiaoxue Zhang, Mingyu Peng, Duwang Su, Pei Han, Yang Zhang

**Affiliations:** Department of Radiology, Qilu Hospital of Shandong University, Jinan, China

**Keywords:** coronary computed tomography angiography, coronary artery disease, ischemic heart disease, fractional flow reserve, strain

**A Corrigendum on**
Correlation between CT-derived fractional flow reserve and myocardial strain in ischemic heart disease patients with single coronary artery stenosis assessed based on CCTA By Ren R, Li W, Zhao Q, Qi C, Zhang X, Peng M, Su D, Han P and Zhang Y (2025). Front Cardiovasc Med. 12:1525807. doi: 10.3389/fcvm.2025.1525807

In the published article, there was an error in Figure 5 as published. The labeling of the sample size in the “Group Patients-Control” section should be (*n* = 114), instead of (*n* = 139).

The corrected Figure 5 and its caption “Diagnostic performance of the CTFFR to recognize impaired myocardial strain in group Patients-control, group LAD-control, group LCX-control and group RCA-control” appear below.

**Figure 5:**
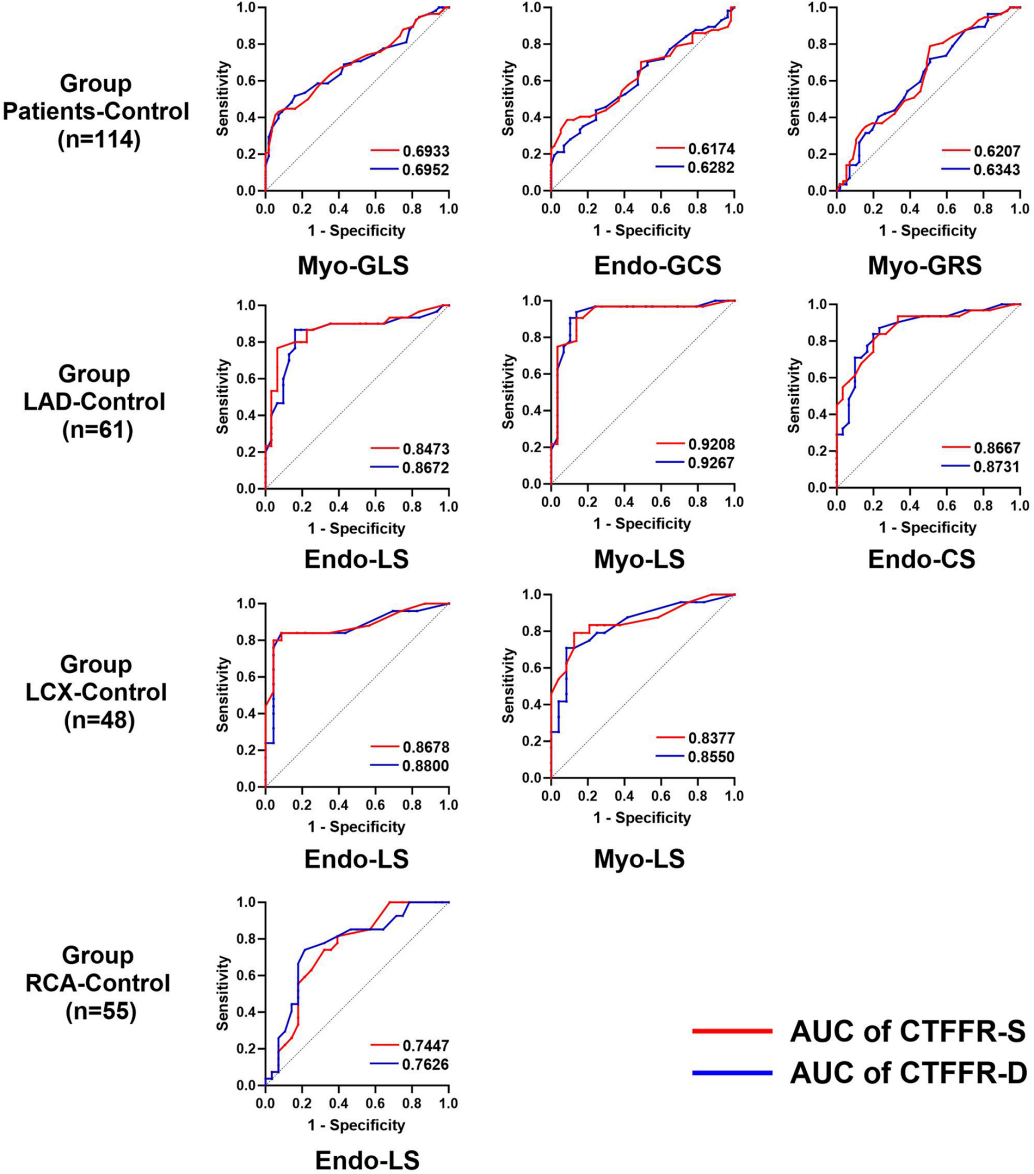
Diagnostic performance of the CTFFR to recognize impaired myocardial strain in group patients-control, group LAD-control, group LCX-control and group RCA-control.

The authors apologize for this error and state that this does not change the scientific conclusions of the article in any way. The original article has been updated.

