# Supplementary material for: Correlation between CT-derived fractional flow reserve and myocardial strain in ischemic heart disease patients with single coronary artery stenosis assessed based on CCTA
Source: Front Cardiovasc Med. 2025 Jan 23;12:1525807. doi: 10.3389/fcvm.2025.1525807 (PMC11798805; doi:10.3389/fcvm.2025.1525807)
Supplement: Supplementary file 1 [file Table1.docx]

Supplementary Table. 1 Sensitivity, specificity and optimal cut-off of ROC curve in Diagnostic performance of the CTFFR to recognize impaired myocardial strain

|  |  | AUC (95% CI) | Optimal Cut-off (Youden Index) | Sensitivity (95% CI) | Specificity (95% CI) |
| --- | --- | --- | --- | --- | --- |
| **Group Patients-Control** | |  |  |  |  |
| Myo-GLS | CTFFR-S | 0.6933 (0.5961 - 0.7906) | 0.775 | 0.5172 (0.3916 - 0.6407) | 0.8393 (0.7219 - 0.9131) |
|  | CTFFR-D | 0.6952 (0.5987 - 0.7917) | 0.735 | 0.4138(0.2963 - 0.5420) | 0.9464 (0.8539 - 0.9854) |
| Endo-GCS | CTFFR-S | 0.6174 (0.5144 - 0.7204) | 0.775 | 0.4386 (0.3177 - 0.5672) | 0.7544 (0.6290 - 0.8477) |
|  | CTFFR-D | 0.6282 (0.5243 - 0.7321) | 0.735 | 0.386 (0.2706 - 0.5157) | 0.9123 (0.8106 - 0.9619) |
| Myo-GRS | CTFFR-S | 0.6207 (0.5179 - 0.7234) | 0.845 | 0.7193 (0.5917 - 0.8192) | 0.4912 (0.3662 - 0.6174) |
|  | CTFFR-D | 0.6343 (0.5323 - 0.7364) | 0.845 | 0.7895 (0.6671 - 0.8753) | 0.4912 (0.3662 - 0.6174) |
| **Group LAD-Control** | |  |  |  |  |
| Endo-LS | CTFFR-S | 0.8473 (0.7411 - 0.9535) | 0.835 | 0.8667 (0.7032 - 0.9469) | 0.8387 (0.6737 - 0.9291) |
|  | CTFFR-D | 0.8672 (0.7686 - 0.9658) | 0.8 | 0.7667 (0.5907 - 0.8821) | 0.9355 (0.7928 - 0.9885) |
| Myo-LS | CTFFR-S | 0.9267 (0.8518 - 1.000) | 0.845 | 0.9063 (0.7578 - 0.9676) | 0.8966 (0.7361 - 0.9642) |
|  | CTFFR-D | 0.9208 (0.8436 - 0.9980) | 0.845 | 0.9063 (0.7578 - 0.9676) | 0.8621 (0.6944 - 0.9450) |
| Endo-CS | CTFFR-S | 0.8667 (0.7733 - 0.9601) | 0.845 | 0.8387 (0.6737 - 0.9291) | 0.8 (0.6269 - 0.9049) |
|  | CTFFR-D | 0.8731 (0.7837 - 0.9625) | 0.835 | 0.8065 (0.6372 - 0.9081) | 0.8 (0.6269 - 0.9049) |
| **Group LCX-Control** | |  |  |  |  |
| Endo-LS | CTFFR-S | 0.8678 (0.7560 - 0.9796) | 0.89 | 0.84 (0.6535 - 0.9360) | 0.913 (0.7320 - 0.9845) |
|  | CTFFR-D | 0.88 (0.7769 - 0.9831) | 0.855 | 0.8 (0.6087 - 0.9114) | 0.9565 (0.7901 - 0.9978) |
| Myo-LS | CTFFR-S | 0.8377 (0.7209 - 0.9544) | 0.855 | 0.7083 (0.5083 - 0.8509) | 0.9167 (0.7415 - 0.9852) |
|  | CTFFR-D | 0.855 (0.7446 - 0.9655) | 0.865 | 0.7917 (0.5953 - 0.9076) | 0.875 (0.6900 - 0.9566) |
| **Group RCA-Control** | |  |  |  |  |
| Endo-LS | CTFFR-S | 0.7447 (0.6122 - 0.8773) | 0.795 | 0.8148 (0.6330 - 0.9182) | 0.6071 (0.4241 - 0.7643) |
|  | CTFFR-D | 0.7626 (0.6316 - 0.8935) | 0.865 | 0.7407 (0.5532 - 0.8683) | 0.7857 (0.6046 - 0.8979) |

Abbreviations: AUC, area under curve; CI, confidence interval; CTFFR-S, computed tomography fractional flow reserve in optimal systole phase; CTFFR-D, computed tomography fractional flow reserve in optimal diastole phase; Myo-GLS, global longitudinal strain of myocardium; Endo-GCS, global circumferential strain of endothelia; Myo-GRS, global radial strain of myocardium; LS, longitudinal strain; CS, circumferential strain; RS, radial strain
